# The cellular landscape of i-motifs: genomic insights, methodological challenges, and the road ahead

**DOI:** 10.1186/s13059-026-04044-8

**Published:** 2026-03-23

**Authors:** Pallabi Sengupta, Nasim Sabouri

**Affiliations:** 1 Department of Medical Biochemistry and Biophysics, Umeå University, 901 87 Umeå, Sweden; 2 Science for Life Laboratory, Umeå University, 901 87 Umeå, Sweden

## Abstract

Non-canonical DNA structures add a dynamic layer of genome regulation beyond the classical double helix. I-motifs, cytosine-rich four-stranded DNA structures, are increasingly recognized as context-dependent regulators of genome function. Recent methodological advances, including i-motif-specific antibodies, in-cell NMR, and genome-wide profiling, have enabled their detection and functional interrogation. Here, we review progress in understanding i-motif formation, stability, and protein interactions, highlighting parallels and contrasts with G-quadruplex structures. We also discuss technical limitations, strategies for improving structure-specific resolution, and future opportunities to integrate biochemical, genomic, and imaging approaches to clarify the biological relevance and therapeutic potential of i-motifs.

## Introduction

The intrinsic structural flexibility of nucleic acids [1] facilitates the formation of many non-canonical structures. Among these, G-quadruplexes (G4s) [2] are well-studied due to their involvement in critical biological processes and potential therapeutic applications, whereas intercalated motifs (i-motifs) [3, 4] are emerging as dynamic four-stranded structures of growing interest in genome biology [5, 6].

**Fig. 1:**
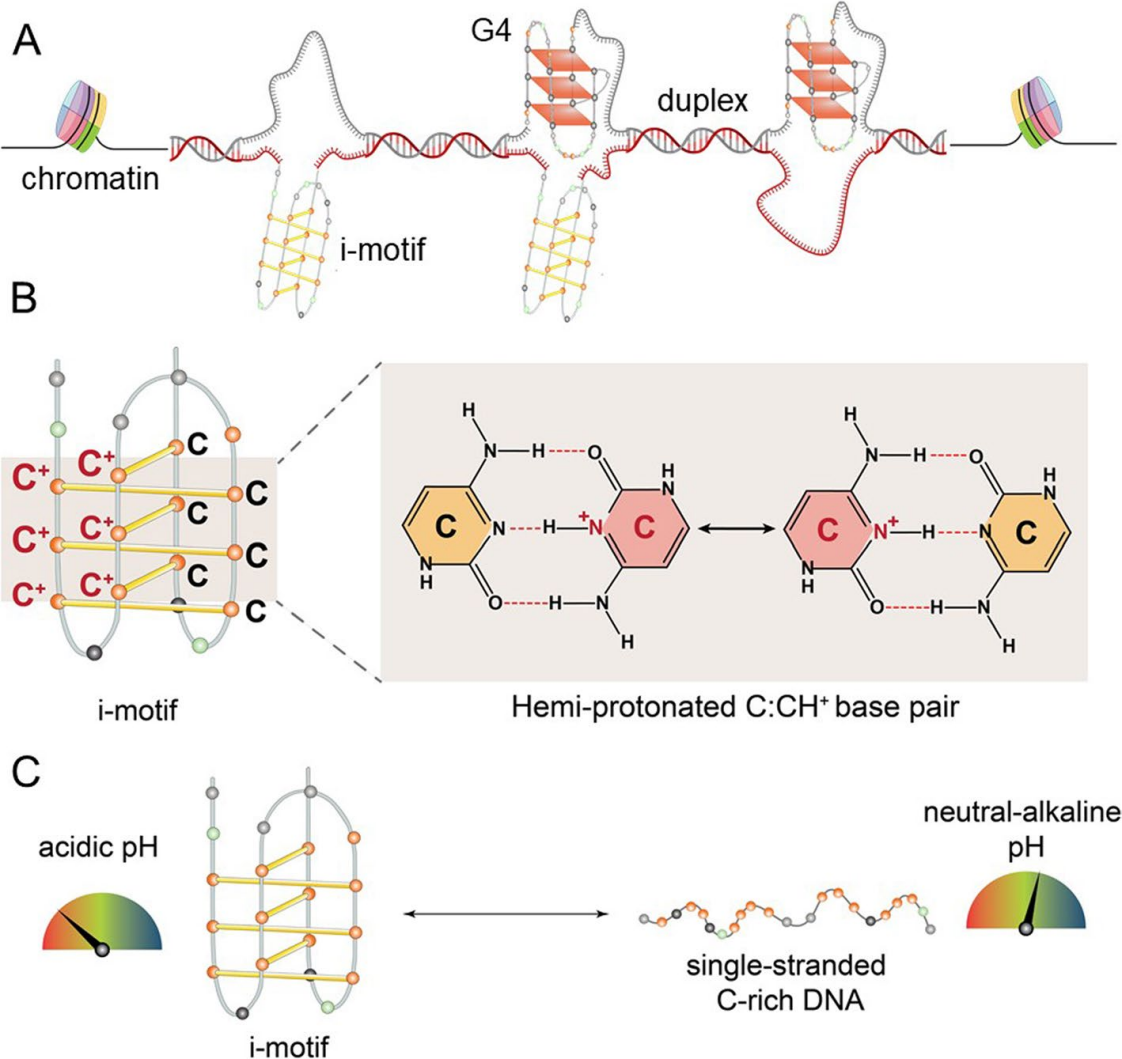
Schematic representation of i-motif DNA structure and its conditions to form. **A** Proposed model of i-motif formation in a genomic context, typically on the C-rich strand opposite to G4-forming sequences. I-motifs and G4s can form transiently and reversibly, often exhibiting mutually exclusive or co-existing dynamics. Created in BioRender. Sabouri, N. (2026) https://BioRender.com/zqjlgut (**B**) In acidic environments, i-motifs readily form through the intercalation of hemi-protonated C:CH⁺ base pairs, driven by protonation of one cytosine in each pair. This folding is pH-sensitive and reversible.** C** Under neutral to alkaline pH in vitro, C-rich sequences predominantly remain unfolded as ssDNA. Created in BioRender. Sabouri, N. (2026) https://BioRender.com/zqjlgut

I-motifs form within cytosine (C)-rich sequences [7], complementary to G4-forming genomic regions (Fig. 1A). They consist of two parallel-stranded duplexes arranged in an intercalated, antiparallel orientation [4] (Fig. 1B), creating two broad major grooves and two exceptionally narrow minor grooves [8]. The latter results in unusually short inter-strand distances and substantial electrostatic repulsion between the negatively charged sugar-phosphate backbones. This intrinsic instability is mitigated by favorable sugar-sugar interactions and hemi-protonated C:CH⁺ base-pairs (Fig. 1B). The localized positive charges introduced by hemi-protonated Cs partially neutralize repulsive forces, thereby stabilizing the core structure [9–11]. Because of these structural constraints, i-motif formation is highly pH-sensitive, readily folding under acidic conditions but unfolding into single-stranded DNA (ssDNA) at neutral pH (Fig. 1C), raising questions about whether the intracellular environment is sufficiently acidic to support their stable formation in vivo [12]. Unlike G4s, which are stabilized by physiological cations and exhibit relatively high stability [13–15], i-motifs are more labile and sensitive to ionic conditions, fluctuations in pH, and temperature [16, 17]. Although an increasing number of experimental studies support i-motifs’ roles in gene regulation, mechanistic understanding remains limited in comparison to G4s. Detecting and manipulating i-motifs in living cells also poses technical challenges, sustaining skepticism regarding their in vivo significance. Their limited characterization in model organisms has further slowed down progress. Thus, while i-motifs represent fascinating structural motifs with potential implications in genome biology, their physiological roles remain an open question.

Nevertheless, the discovery of i-motif-specific antibodies [18, 19] has greatly advanced cellular studies by enabling the detection of putative i-motifs in mammalian nuclei and highlighting potential roles in gene regulation. Although it is now clear that non-physiological low temperatures can promote i-motif formation, and thus complicate the question of whether they form under true physiological conditions, there is evidence that indicates that their distribution differs between cancer and normal cells [20], suggesting that non-thermal cellular factors could contribute to the conditions under which i-motifs form or persist. Their pH sensitivity and reversible folding also make them attractive candidates for novel molecular switches or biosensors [21–24]. As recent studies have increasingly uncovered the impact of cellular heterogeneity, local pH variations, and nuclear compartmentalization [25], the contextual relevance of i-motifs has become clearer. Thus, even as pH and temperature-dependent stabilization presents interpretational challenges, i-motifs are increasingly recognized as context-dependent, responsive DNA elements with significant potential in both genome biology and biomedical applications.

### Advances in the cellular research of i-motifs, including strengths and limitations

Since their discovery approximately three decades ago, a major turning point in this field has been the development of iMab, an antibody fragment that binds C-rich DNA regions with high preference for i-motif structures [18]. Following this breakthrough, iMab has been employed across diverse experimental settings to investigate the presence, dynamics, and regulatory functions of i-motifs within cells. However, a recent study argued over non-specific interactions of iMab with C-rich ssDNA stretches under acidic conditions, independent of i-motif folding and that iMab promotes unfolding of pre-folded i-motif structures in vitro [26]. This study highlights the potential limitations of iMab for certain C-rich sequences and underscores the need for careful interpretation of iMab-based detection including proper controls, particularly in relation to the physiological formation of i-motifs. However, it should also be acknowledged that these findings are observed within in vitro set-up using short synthetic sequences and may not fully recapitulate the crowded nuclear environment. Despite these caveats, iMab‑based studies have revealed highly reproducible overlapping patterns across independent studies, suggesting biological relevance, reigniting interest in their potential biological significance. Below, we summarize several key analyses, including their strengths and limitations.

### Immunofluorescence

**Fig. 2:**
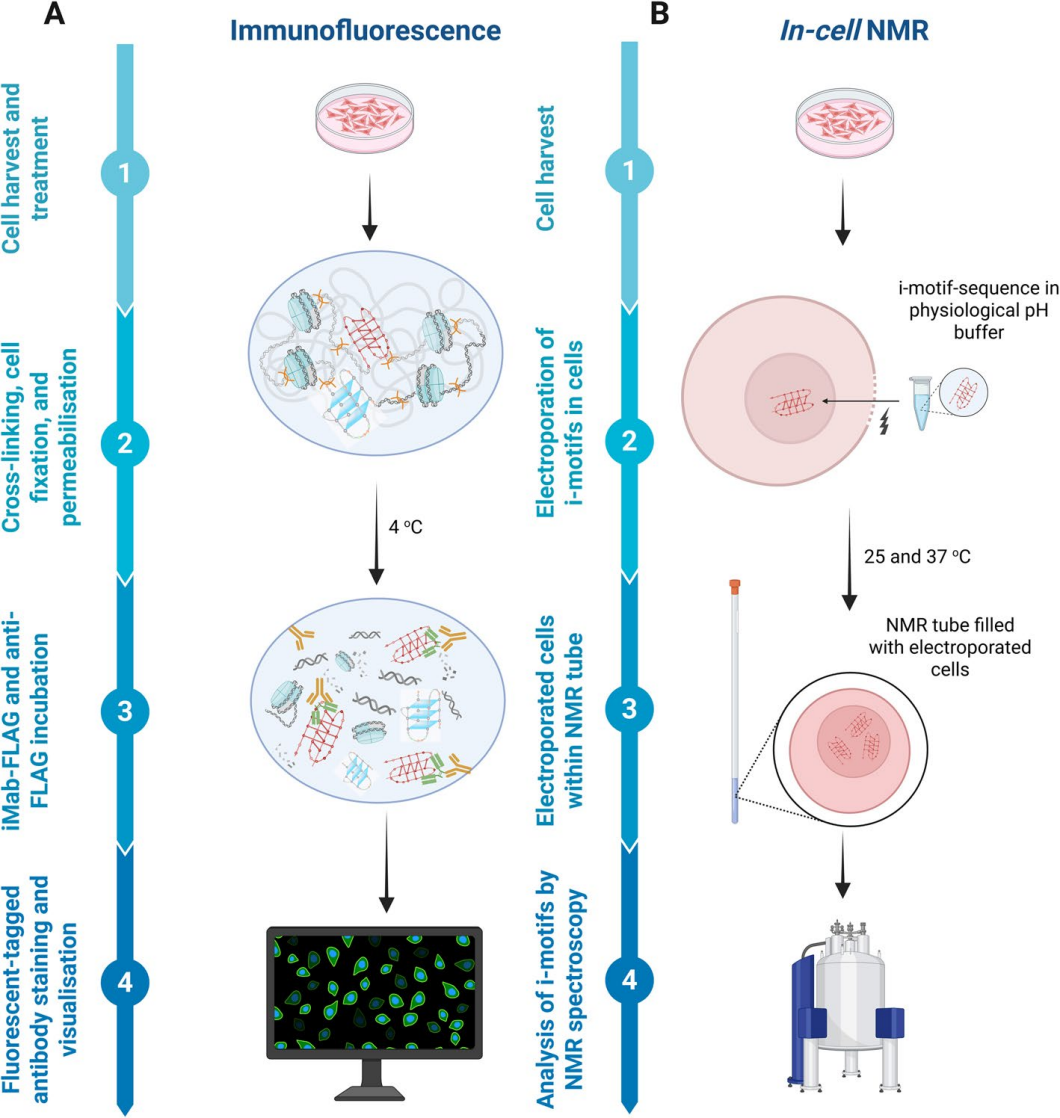
Progress in cellular research in i-motif field. **A** Immunofluorescence studies involving incubation of iMab antibody in cross-linked cells upon synchronization or hypercapnia at 4 °C – showing i-motifs in the nuclei. **B** In-cell NMR method that involves electroporation of synthetic i-motif-forming oligonucleotides folded in pH 7.4 inside cells followed by investigation of i-motif formation by 1D ^1^H NMR spectroscopy. Created in BioRender. Sabouri, N. (2026) https://BioRender.com/rzp9ift

One of the earliest and widely adopted applications of iMab is the immunofluorescence-based detection of putative i-motifs recognized by iMab (Fig. 2A) [18]. These studies, which were performed under various cellular conditions, including distinct cell cycle phases and controlled modulations of the intracellular pH through CO₂ levels, reveal dynamic spatiotemporal patterns of putative i-motifs. These loci appear and resolve in a cell cycle–dependent manner, reciprocally to G4s [27, 28]. This trend aligns with findings from biophysical approaches, including single-molecule-based assays, which demonstrated that G4s and i-motifs generally form in a mutually exclusive fashion within certain oncogene promoters [29, 30]. In a few cases, where both structures coexist, their offset positioning on complementary strands alleviates steric hindrance [31, 32]. Furthermore, a reduced CO₂ supply, which lowers the endogenous pH, significantly increases the iMab signal, mirroring the acidic conditions required for i-motif formation in vitro [18].

Moreover, immunofluorescence studies [18] have demonstrated significant colocalization of iMab signal with telomeric repeat-binding factor 2 (TRF2), a known negative regulator of telomere length [33], and with E12/E47 transcription factors (TFs) that bind consensus E-box motifs (CANNTG; N denotes any nucleotide) within promoter or enhancer regions [34]. These findings align with bioinformatically predicted and systematically studied i-motif-forming sequences [35–37] in the genome that typically conform to a C-run/loop architecture of four C-tracts of length 3–5 separated by three loops (approximately 1–7 nucleotides), summarized by the generic pattern C_3–5_N_1–7_C_3–5_N_1–7_C_3–5_N_1–7_C_3–5_, with loops often enriched in A/T*.* Systematic studies on i-motif sequences highlight that longer C-tracts increase the i-motif stability, and that the total spacer length is not as critical factor as it is for G4 structures [37]. The described generic pattern is consistently recovered across current prediction tools, including flexible run/loop search algorithms (*e.g.*, G4-iM Grinder [38]), hybrid rule-based and machine-learning approaches (*e.g.*, iM Seeker [36]), and Position-Specific Similarity Matrix (PSSM)-based high-confidence motif sets [39], which differ in scoring schemes but converge on this same four-tract, short-loop C-rich architecture. Thus, while no single unique sequence definition exists, a closely related family of C-tract motifs constitutes the consensus i-motif–forming pattern. In addition, they are supported by cell-based and in vitro assays implicating specific i-motifs (*e.g.*, from *c-MYC* and *BCL2* promoters) [40, 41], in transcription regulation. Recent genome-wide sequencing studies reinforce the preferential localization of putative i-motifs to conserved genomic regions [20, 42], particularly telomeres and gene promoters, suggesting potential roles in transcriptional regulation and telomere maintenance.

An important consideration is that immunofluorescence studies, which rely on fixed cells at low temperatures (typically 4 °C), may artificially promote i-motif stabilization. As a result, observations of iMab binding at these temperatures may overestimate i-motif formation under physiological conditions (~ 37 °C). Therefore, while these methods provide valuable spatial and temporal insights, they can be further strengthened by complementary live-cell approaches under physiological temperature. A recently developed nanobody, called iMbody, opens new possibilities in this direction [19]. iMbody has not yet been widely utilized, but it has so far been shown to be suitable for enzyme-linked immunosorbent assay (ELISA) and immunofluorescence studies. Its small size (17.3 kDa) raises the possibility that iMbody could be modified and expressed as an iMbody-fluorescent fusion protein for live-cell imaging, potentially enabling visualization of i-motif dynamics in cells under physiological conditions.

The methodological limitations of immunofluorescence, particularly the potential stabilization of i-motifs at low temperatures and the possibility that such conditions may increase the number of detectable sites, suggest careful analysis and consideration. Nonetheless, several features of this approach suggest that at least a subset of the observed signals likely reflect structures present prior to cell fixation. For instance, the dynamic appearance of iMab-signal during particular cell cycle phases and in response to intracellular pH shifts suggest regulated i-motif formation rather than fixation artefacts. Also, nuclear foci detected by iMab are abolished upon DNase treatment, confirming their DNA origin and antibody specificity. The absence of signals in the cytosol or nucleolus further supports this conclusion and aligns with biophysical evidence that RNA is unlikely to form i-motifs under physiological conditions, owing to its backbone rigidity, steric hindrance from the 2'-hydroxyl group, and its preference for an A-form helix–incompatible i-motif geometry [43]. Finally, their colocalization with biologically relevant proteins, such as TRF2, underscores their functional importance rather than their incidental occurrence. Although these observations do not eliminate the potential temperature and fixation-based artefact mentioned earlier, their convergence across multiple experimental contexts indicates that immunofluorescence can provide meaningful insight into the occurrence and distribution of i-motif–associated regions in cells. Thus, while careful interpretation and appropriate experimental controls are essential, this methodology continues to serve as a valuable tool for exploring the potential formation of i-motifs in the cellular environment.

### *In-cell* NMR

*In-cell* NMR is a direct approach for investigating i-motif formation within living cells [12, 44] (Fig. 2B). In this method, synthetic oligonucleotides containing known i-motif-forming sequences are electroporated into cultured cells. The electroporated cells are then loaded into NMR tubes, enabling real-time analysis of i-motif formation under near-physiological conditions. Nuclear localization and cellular uptake are validated by fluorescently-tagged sequences visualized through confocal microscopy, while flow cytometry before and after NMR measurements ensures cell viability. Following NMR analysis, the cells are lysed, and the internalized DNA is re-examined under acidic conditions to verify its capacity to refold into i-motifs ex vivo. Interestingly, only a subset of sequences, capable of forming i-motifs in vitro adopt this structure in cells, underscoring the importance of intracellular context in dictating folding behavior.

As this approach relies on short oligonucleotides, it does not capture the chromatin environment, which plays an important role in shaping DNA topology and accessibility. Tang et al*.* showed in *Bombyx mori* testis that i-motif foci are nearly twice as abundant during interphase as they are during mitosis, highlighting how relaxed chromatin can promote i-motif formation [45]. However, it can be integrated with epigenomic profiling by combining i-motif sequencing with chromatin state markers to reveal how the genomic context or disease-associated mutations influence folding. Electroporated oligonucleotides may also increase the risk of artefactual folding or degradation [46, 47] (*e.g.*, TREX1 (3′-repair exonuclease 1)) [48], or sequestration by proteins [49] that obscure signals. Despite these considerations, *in-cell* NMR enables real-time and high-resolution structural analysis at physiological temperature and ionic conditions in living, intact cells. Orthogonal validations, including confocal microscopy for nuclear localization and flow cytometry for cell viability, reinforces the biological relevance of the detected structures. This method can also be paired with fluorescence in situ hybridization (FISH) to map specific i-motifs spatially, similar to the CRISPR-dCas9-assisted FISH strategies, previously used in G4 research [50, 51]. Furthermore, *in-cell* NMR provides the ability to analyze cells upon cell cycle synchronization, thereby enhancing the understanding of context-dependent i-motif formation. Using TREX1-deficient cells or cell-types with low nuclease activity (*e.g.*, iPSCs (induced pluripotent stem cells)) may increase oligonucleotide stability and signal clarity [48]. Thus, *in-cell* NMR offers a compelling and highly specific strategy for probing the real-time dynamics of i-motif structures within living cells.

### High-throughput genome sequencing

**Fig. 3:**
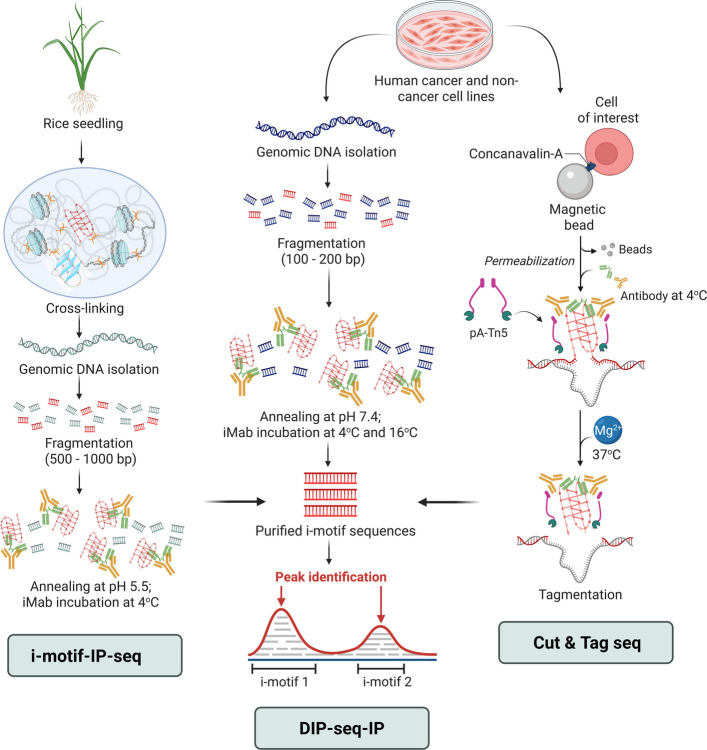
Advances in sequencing studies on i-motif distribution in the genomes of different organisms and cell-types. I-motif-IP-seq in rice seedlings involves isolation and fragmentation of genomic DNA from rice seedlings, and their annealing at pH 5.5 followed by iMab incubation at 4 °C. DIP-sequencing-IP involves genomic DNA isolation and fragmentation from human cell lines without cross-linking, followed by annealing at pH 7.4, antibody incubation at 4 °C and 16 °C. CUT&Tag sequencing involves immobilization of live human cells on concanavalin A beads followed by their permeabilization without cross-linking before iMab antibody incubation. Following antibody binding, genomic DNA was accessed via pA-Tn5 transposase-mediated tagmentation, enabling the identification of i-motif-forming regions directly within native chromatin. Created in BioRender. Sabouri, N. (2026) https://BioRender.com/xshpgs9

While immunofluorescence studies provide broad visualization of iMab/iMbody-enriched regions and *in-cell* NMR offers sequence-specific insights into i-motif folding within cells, sequencing-based approaches have expanded the field by enabling genome-wide mapping of iMab-enriched putative i-motif–forming regions in rice seedlings and human cells. These studies employ diverse methodologies and experimental conditions, each with distinct considerations (Fig. 3).

#### i-motif-IP-seq in rice seedlings

This study presents the first genome-wide mapping of iMab-enriched C-rich regions in a non-human organism, and remains the only detailed investigation of i-motif formation in plants. In this study, rice seedlings are cross-linked at pH 8.0, followed by genomic DNA extraction [52], fragmentation, and reconstitution in a pH 5.5 buffer with a molecular crowding agent. The DNA is then incubated with iMab at low temperature and the immunoprecipitated material was sequenced (Fig. 3). The resulting maps showed enrichment of putative i-motif–forming sequences in promoters and UTRs (Untranslated regions), and depletion in exons and distal exogenic sites—patterns broadly consistent with human datasets. These regions also display differential histone methylation patterns: gene bodies or terminal putative i-motif-forming regions are less enriched in H3K27me3, while promoter-associated putative i-motifs show no enrichment in H3K9me3. Beyond transcriptional regulation, they also influence transposable element dynamics among rice subpopulations, with implications for plant biology and biotechnology.

However, the absence of chromatin, together with the use of highly acidic reconstitution conditions and low-temperature antibody incubation for extended time, may favor i-motif folding in vitro. Additionally, recent studies suggesting potential non-specific binding of iMab to ssDNA [26] have raised caution regarding possible artefactual signals. Nevertheless, this study offers a foundational plant genome-wide map of putative i-motif-forming sequences, extending i-motif research beyond human systems.

#### DIP-IP-seq in human cell lines

This study examined putative i-motif-forming regions in three human cell lines, MCF-7, U2OS, and HEK293T. Fragmented genomic DNA was reconstituted and annealed at physiological pH (7.4), followed by iMab incubation at both 4 and 16 °C to evaluate temperature effects on iMab-bound signals [53] (Fig. 3). Approximately 96,086 iMab-peaks were detected at 4 °C, but only 55,080 persisted at 16 °C, highlighting the inherent temperature sensitivity of potential i-motif-forming sites. These findings are consistent with those of *in-cell* NMR, which have shown the temperature-sensitive behavior of well-characterized i-motifs (*e.g.*, telomeric and *PDGFa*), that are detectable at 20 °C but disappear at 37 °C. Interestingly, certain C-rich sequences, such as *RAD17*, retained detectable signals at 37 °C, underscoring sequence- and context-dependent stability.

Approximately 53,000 putative i-motif-forming regions are consistently observed across all three cell lines, indicating conserved genomic hotspots, while others exhibit cell-type specificity. Furthermore, putative i-motif-forming loci are positively correlated with euchromatic marks and negatively with heterochromatic marks, mirroring the findings from rice seedlings. These loci are preferentially enriched within transcriptionally active regions, particularly in promoters and 5′-UTRs of genes upregulated during G_0_/G_1_ phase. They are also enriched in TAD (Topologically Associating Domain) boundaries, which are CTCF (CCCTC binding factor) hotspots, known to recognize C-rich sequences in vitro, implicating that these structures might contribute to higher-order genome organization [54].

#### CUT&Tag sequencing in human cell lines

This study has been conducted in HEK293T and WDLPS cell lines, offering a methodologically distinct approach that interrogates chromatin-associated C-rich regions in permeabilized, live cells [20]. Unlike previous studies relying on fixation, or naked genomic DNA, live cells are immobilized on concanavalin A beads, gently permeabilized, and incubated with iMab before pA-Tn5 transposase-mediated tagmentation (Fig. 3). Despite these advantages, it still relies on low-temperature incubation steps, and therefore identifies C-rich regions prone to i-motif formation rather than confirmed i-motifs. Nevertheless, parallel mapping of iMab and H3K4me3 is performed in the same samples, enabling direct comparison with active chromatin marks. Consistent with previous sequencing and immunofluorescence assays, putative i-motif-forming regions are mostly abundant in the gene promoters, open chromatin and show substantial overlap with R-loop regions, suggesting their mechanistic interplay in transcription regulation. Conversely, i-motif-forming regions within R-loops may modulate chromatin accessibility or TF recruitment. Zanin et al*.* further reported substantial overlap between BG4 and iMab-bound peaks, alongside exclusive peaks for each antibody, providing evidence that G4- and i-motif-forming regions may fold independently as well as interdependently in cells. Overall, this represents the most physiologically relevant genome-wide mapping to date, while still subject to the inherent constraints of antibody-dependent detection and low temperatures.

### Comparative insights from sequencing studies and future directions

Sequencing studies involve different experimental approaches, which inevitably yield variable maps for i-motif-prone genomic regions, but additional biological and technical factors also contribute to these differences. Different cell-types display distinct dynamics of nucleic acid secondary structures. Balasubramanian’s group has reported that cancer and stem cells harbor more G4s than non-transformed cells, due to their chromatin packaging and remodeling [55]. Since G4s and i-motifs form in complementary regions, and considering the pH sensitivity and transient nature of i-motifs, their distribution is expected to vary across different cell-types. In particular, cancer cells, which are metabolically hyperactive and often overexpress oncogenes [56, 57], may provide favorable contexts for transient C-rich folding events during transcription.

Cell-cycle–dependent changes also influence i-motif propensity. For instance, in G_1_, the genome is relatively decondensed, whereas S phase involves DNA unwinding and replication-associated helicase activity that destabilizes i-motifs. Intracellular pH (pHi) also fluctuates, decreasing at G_1_/S that might favor i-motif stabilization, but increasing at S and G_2_, reducing its stability [58]. Since ChIP-seq and CUT&Tag are typically performed on asynchronous cultures, the resulting maps represent ensemble averages rather than phase-specific snapshots, masking these transient phase-specific folding events. Even within the same population, individual cells differ in metabolism, ion concentrations, and local chromatin remodeling, all of which may affect i-motif propensity. Temperature is another important variable: lower temperatures stabilize i-motifs, while even modest increases accelerate unfolding. Notably, the immunoprecipitation-based protocols involve processing steps at 4 °C, while DIP-IP was performed at 16 °C, it is still much below physiological temperatures, which may stabilize otherwise transient structures in cells. Despite the limitations, current approaches represent important methodological steps that have progressively advanced the field—from early acidic pH-based assays to physiological pH, from fixed cells to live-cell sequencing, and from permissive to progressively higher temperatures. These advances highlight both the pioneering nature of the work and the practical constraints imposed by the transient, pH- and temperature-sensitive nature of i-motifs. Moreover, these low-temperature datasets still provide a useful ‘positive-control’ landscape of maximally foldable regions since regions that fail to fold even under permissive conditions are unlikely to form i-motifs in vivo.

Looking ahead, several strategies may strengthen the interpretation of sequencing results. Cross-validation using multiple antibodies (iMab, iMbody) can minimize assay-specific bias. Minimizing low-temperature steps, for example by shortening 4 °C incubations or conducting workflows at controlled physiological temperatures can mitigate temperature-associated artefacts. In such cases, developing antibody fragments or chemical probes validated for stability and specificity at 37 °C will allow more physiologically relevant mapping. Complementing antibody-based methods with orthogonal methods, including *in-cell* NMR or temperature-controlled smFRET (single-molecule Förster resonance energy transfer) can provide direct confirmation of folding states. SmFRET is advantageous because it can detect folding properties at very low concentrations, comparable to those in cells, and monitor single-molecule dynamics in real time, revealing conformational heterogeneity. However, it does not capture the effects of molecular crowding or the complex cellular environment like *in-cell* NMR, and requires site-specific labeling that may perturb native structures, and is limited to in vitro conditions. The use of mutated C-rich control sequences can help to distinguish genuine structures from non-specific interactions. Combining these data sets with machine learning models may refine predictive algorithms for identifying i-motifs genome-wide. Time-resolved profiling across different pH conditions or cell cycle phases would also better capture their transient nature. Comparative mapping with G4s may further improve site identification, given their complementary distribution. Finally, emerging tools, such as selective chemical probes for competitive binding studies, and CRISPR-based disruption of candidate loci offer functional validation. Collectively, these approaches represent a robust framework for dissecting i-motif biology within complex nuclear environments (Table 1).

**Table 1:** Future directions for cellular research on i-motifs

Approach	Description
Use multiple antibodies	Compare results across iMab and iMbody alongside BG4 and development of additional i-motif antibodies
Integrate multiple methods	Combine antibody-based techniques with physicochemically direct methods like in-cell NMR or FRET
Use mutated sequences	Incorporate mutated C-rich sequences from the genome that can’t form i-motifs to assess nonspecific binding
Time-Resolved Profiling	Perform ChIP-seq across different pH conditions or cell cycle phases to detect dynamics indicative of folded versus unfolded states
Compare with G4 Profiling	Compare complementarity between G4 and i-motif mapping (both structure and location) to filter ambiguous sites
Chemical probes	Use small molecule ligands highly specific for i-motifs for competitive binding assays
CRISPR-based disruption	Target candidate i-motif hotspots and assess changes in binding and function upon targeted disruption
I-motif-protein interactions	Genomic studies on specific i-motif-binding proteins to enhance mechanistic insights

### Dynamic regulation of i-motifs by intracellular and environmental conditions

pHi undergoes dynamic fluctuations under various conditions that provide a compelling biophysical basis for i-motif dynamics. Under in vitro conditions, i-motif folding follows a kinetic partitioning mechanism, resulting in very slow kinetics [59]. However, within cells, i-motif dynamics are shaped by a complex interplay of regulatory proteins, TFs, chromatin context, and cellular stimuli, and the precise intracellular folding kinetics of i-motifs are not yet understood. Studies across diverse biological systems demonstrate that cellular conditions can profoundly alter biomolecular folding kinetics; for instance, molecular crowding alone can accelerate nucleic acid folding [60]. Consequently, in vitro behavior cannot be directly extrapolated to the intracellular environment. Future work combining *in-cell* NMR in live cells, genome-integrated fluorescent i-motif reporters, and/or time-resolved chromatin immunoprecipitation with rapid fixation will be essential for directly probing the timescales and regulatory mechanisms governing i-motif folding in vivo.

#### Cancer

Cancer cells exhibit a reversed pH gradient [61], maintaining an elevated pHi (≥ 7.4) despite a lower extracellular pH (pHe) (≤ 7.0) [62–66]. This hallmark feature [67] arises from dysregulated activity of proton transporters and ion exchangers (*e.g.*, Na⁺/H⁺ exchanger (NHE), H⁺-ATPases). By extruding protons, these systems counteract the acidifying effects of aerobic glycolysis (Warburg effect), which generates lactate that acidifies the tumor microenvironment [68]. Chronic extracellular acidosis suppresses acid-loading transporters such as anion exchanger 2, which imports HCO_3_⁻ in exchange for intracellular Cl⁻ to promote cytoplasmic acidification [69]. Using this pH-responsiveness, i-motif-conjugated inorganic nanoparticles have been used as nucleic acid sensors and therapeutic carriers [70, 71]. Anticancer drugs (*e.g.*, doxorubicin) could be loaded into i-motif-based nanomaterials or exosomes enabling pH-triggered release in acidic tumor microenvironments [72–74].

#### Stress

Various physiological and environmental stressors induce intracellular acidification. Osmotic stress, heat shock, and hypercapnia (elevated CO_2_ levels) reduce pHi [75–77], while oxidative stress–often accompanied by glutathione depletion–impairs Na^+^/H^+^ antiporter activity, reducing cellular capacity to recover from acid loads [78]. Ischemia and hypoxia decrease both pHi and pHe, particularly in neural tissue [79], with pre-ischemic hyperglycemia further exacerbating intra- and extracellular acidosis during ischemic episodes [80]. Such pH and redox imbalances directly influence DNA structural dynamics. For instance, immunofluorescence studies have revealed increased putative i-motif formation following intracellular acidification by hypercapnia [18]. Similarly, oxidative stress enhances G4 abundance in zebrafish embryos and cancer cells [81], while transgenic zebrafish models of rhabdomyosarcoma exhibit elevated G4 levels compared with healthy tissue, as well as increased sensitivity to oxidative stress [82]. Together, these findings suggest that stress-induced changes in cells create permissive niches for non-canonical DNA structures.

#### Aging

Aging profoundly influences pHi regulation [83]. In young cells, pHi homeostasis is maintained by the coordinated action of plasma membrane P-type H^+^-ATPases and lysosomal/vacuolar V-type H^+^-ATPases, which balance proton fluxes across cellular compartments and maintain organellar acidity. With age, this coordination deteriorates [84]. In aged rat hippocampal neurons, impaired Na^+^/K^+^-ATPase and Na^+^/H^+^-exchangers functions accumulate cytosolic protons, lowering pHi. Aging also compromises cellular buffering capacity, making cells more vulnerable to acid–base disturbances. Oxidative stress during aging inhibits the proton-pumping function of V-type H^+^-ATPase, thereby reducing lysosomal/vacuolar acidification. Together, these alterations contribute to a dysregulated pHi in aged cells, with downstream implications for organellar function, metabolic activity, and stress responses [83, 85]. Although impact of aging on i-motifs is not directly investigated yet, recent studies have shown that aging is associated with accumulation of G4s within cells and mouse and human brain tissues [86].

#### Viral infection

Viral infections perturb pHi that varies with the virus type, host cell characteristics, and the stage of infection [87–90]. In early stages of influenza or Sindbis infection, cytoplasmic pH is lowered [91, 92]. Certain viruses manipulate ion transporters to increase proton efflux, thereby raising pHi [87]. Host cells respond to these pHi fluctuations through mechanisms, such as upregulating proton-exporting transporters, to restore homeostasis. Although direct evidence of viral infection altering host i-motif formation is lacking, studies have shown that the *HIV-1* LTR promoter itself adopts an i-motif, which is stabilized by the host protein, hnRNP-K to stall viral transcription machinery [93]. Also, putative i-motifs recently identified in promoters of human alphaherpesviruses are highly conserved, suggesting biological functions [94].

#### Epigenetic modification

Epigenetic modifications strongly influence the stability and dynamics of i-motifs. Among these, 5-methylcytosine (5mC) generally stabilizes i-motifs, whereas 5-hydroxymethylcytosine (5hmC), 5-formylcytosine (5fC) and 5-carboxylcytosine (5caC) tend to modulate their stability in a sequence- and position-dependent manner. The stabilizing effect of 5mC arises from enhanced base stacking and increased hydrophobicity, which elevate melting temperatures and permit i-motif folding at near-physiological pH. These stabilizing effects are more evident when 5mC is positioned in the central C-tracts. In contrast, 5hmC introduces a polar hydroxymethyl group that disrupts stacking and hydration within the intercalated core, while 5fC and 5caC, both electron-withdrawing, lower the proton affinity of cytosine N3, weakening the hemi-protonated C:CH⁺ pair, essential for i-motif formation. The negative charge of 5caC further introduces electrostatic repulsion within the i-motif core. Wright et al*.* have systematically investigated these effects in human telomeric i-motif sequences [95], showing that 5mC generally raises the transition pH and thermal stability, whereas 5hmC, 5fC and 5caC exert more variable, context-dependent modulation. The influence of each modification is highly dependent on the position of specific Cs within the sequence, underscoring the relationship between epigenetic marks and i-motif stability [96]. Importantly, methylome comparisons between MCF-7 (cancerous) and MCF-10A (non-cancerous) breast epithelial cells revealed that i-motif-forming sequences capable of folding near neutral pH are disproportionately enriched for DNA methylation, suggesting that epigenetic modifications may act as a regulatory mechanism to stabilize i-motifs in physiologically active genomic regions, particularly in disease contexts.

#### G4 formation on the complementary strands

Although G4s and i-motifs are generally viewed as mutually exclusive when located on complementary strands, recent evidence indicates that their relationship is more context-dependent [97]. Steric constraints arising from the sugar–phosphate backbones, which form four grooves of distinct dimensions in both structures, led to the idea that simultaneous folding of a G4 on one strand and an i-motif on the opposite strand would be structurally incompatible, and that their simultaneous appearance is possible only when they are in significantly distant offset position to negate the steric repulsion [29]. This was supported by many in vitro studies, including single molecule studies, and by cell-cycle–resolved studies showing reciprocal formation of G4s and i-motifs at several oncogene promoters in different phases [18, 93, 94].

However, several lines of evidence challenge a strictly mutually exclusive model [98–100]. G4s are considerably more stable than i-motifs under physiological conditions, and i-motif forming sequences mostly remain unfolded, frequently appearing as bulged or flexible single-stranded elements, as illustrated by the co-crystal structure reported by Warner et al., in which a stable G4 forms on one strand of a duplex while the complementary C-rich strand does not adopt an i-motif but instead bulges outward [101]. *In-cell* NMR studies also indicate that many i-motif–forming regions remain unfolded and are bound by proteins or TFs, thereby restricting their ability to fold back [12]. These unfolded or protein-occupied states are biologically meaningful, as failure to properly resolve or unwind such structures can contribute to replication stress, deletions, and genomic instability [102]. While G4s and i-motifs require distinct ionic and pH conditions, supporting the theory of mutual exclusion, mechanical stress affects both structures by regulating the kinetics of the duplex ↔ tetraplex equilibrium rather than directly stabilizing one species [103]. Under certain torsional or crowded conditions, this creates a permissive environment where both G4s and i-motifs can fold or unfold simultaneously. Consistent with this, recent smFRET and replication assays have observed signatures of concurrent G4 and i-motif formation at specific loci, with their stabilities tightly coupled. Furthermore, the oncogenic splicing factor, PCBP2, was identified as an i-motif–interacting protein that destabilizes i-motifs, and consequently the facing G4s, facilitating replication past these otherwise persistent structures [104].

Nevertheless, the temporal window for i-motif folding in vivo appears to be narrow, and their highly dynamic nature makes simultaneous detection experimentally challenging. Recent immunoprecipitation and sequencing-based findings indicate that while reciprocal folding during the cell cycle is common, coexistence at specific genomic regions does occur, highlighting that the spatiotemporal dynamics of these structures are context dependent. Future work is needed to identify the regulatory proteins and stimuli that stabilize, rather than unfold, i-motifs and to define how these factors modulate their coexistence with G4s in vivo.

#### Chromatin context

Chromatin architecture and its associated epigenetic modifications strongly influence i-motif formation. Histone acetylation, typically linked to transcriptionally active and open chromatin, may enhance i-motif accessibility by increasing DNA exposure, whereas repressive histone marks and chromatin compaction can restrict folding at C-rich loci. CpG methylation stabilizes i-motifs by promoting favorable base-stacking and protonation dynamics, with cancer-associated methylation patterns and CpG-rich repeats favoring i-motif formation under physiological pH. Genomic mapping studies have further revealed that i-motif-forming sequences often overlap with binding sites for chromatin remodelers (*e.g.*, MTA2, SUZ12) and TFs (*e.g.*, E4F1, E2F8, SP1, CREB1), but rarely with DNA repair factors. This suggests that i-motifs preferentially localize within transcriptional and epigenetic regulatory hubs rather than repair-associated domains. Sequence context also modulates i-motif dynamics. Longer CCG repeats, as seen in the upstream of *FMR1* gene, exhibit a higher propensity to form hairpins that are particularly susceptible to CpG methylation. These structures can mediate transcriptional silencing through methylation-mediated gene repression but also contribute to genomic instability during replication [105].

Taken together, i-motif formation emerges as the product of an intricate interplay between many factors creating a dynamic yet tightly regulated structural landscape within the genome.

### Protein interaction studies with i-motifs

Most i-motif–binding proteins have been identified in vitro, and their physiological relevance remains unclear. A major caveat is the intrinsic pH sensitivity of i-motifs, which complicates replication of nuclear conditions in in vitro assays and raises uncertainties over whether proteins reported to bind i-motifs interact with folded structures or with their single-stranded counterparts. Another complication arises from the pH sensitivity of proteins themselves, many of which exhibit reduced activity or stability under the acidic conditions, thereby confounding the interpretation of their interactions in vitro. Nevertheless, the transient folding of putative i-motifs within nuclei suggests that proteins can facilitate or stabilize C-protonation through several mechanisms.

Proteins may influence i-motif dynamics indirectly by recruiting chromatin remodelers or modifying enzymes, thereby altering DNA accessibility, local ionic strength, or cytosine chemistry (*e.g.*, 5mC, 5hmC), all of which impact folding behavior. Supporting this view, recent proteomic studies have identified 1,329 candidate proteins with i-motif-binding potential [106]. Some of them bind to 5mC (ZBTB [107, 108] and MBD family proteins [109]) or 5hmC (UHRF2 [110]), suggesting that recognition of C modifications and i-motifs may be functionally linked.

**Fig. 4:**
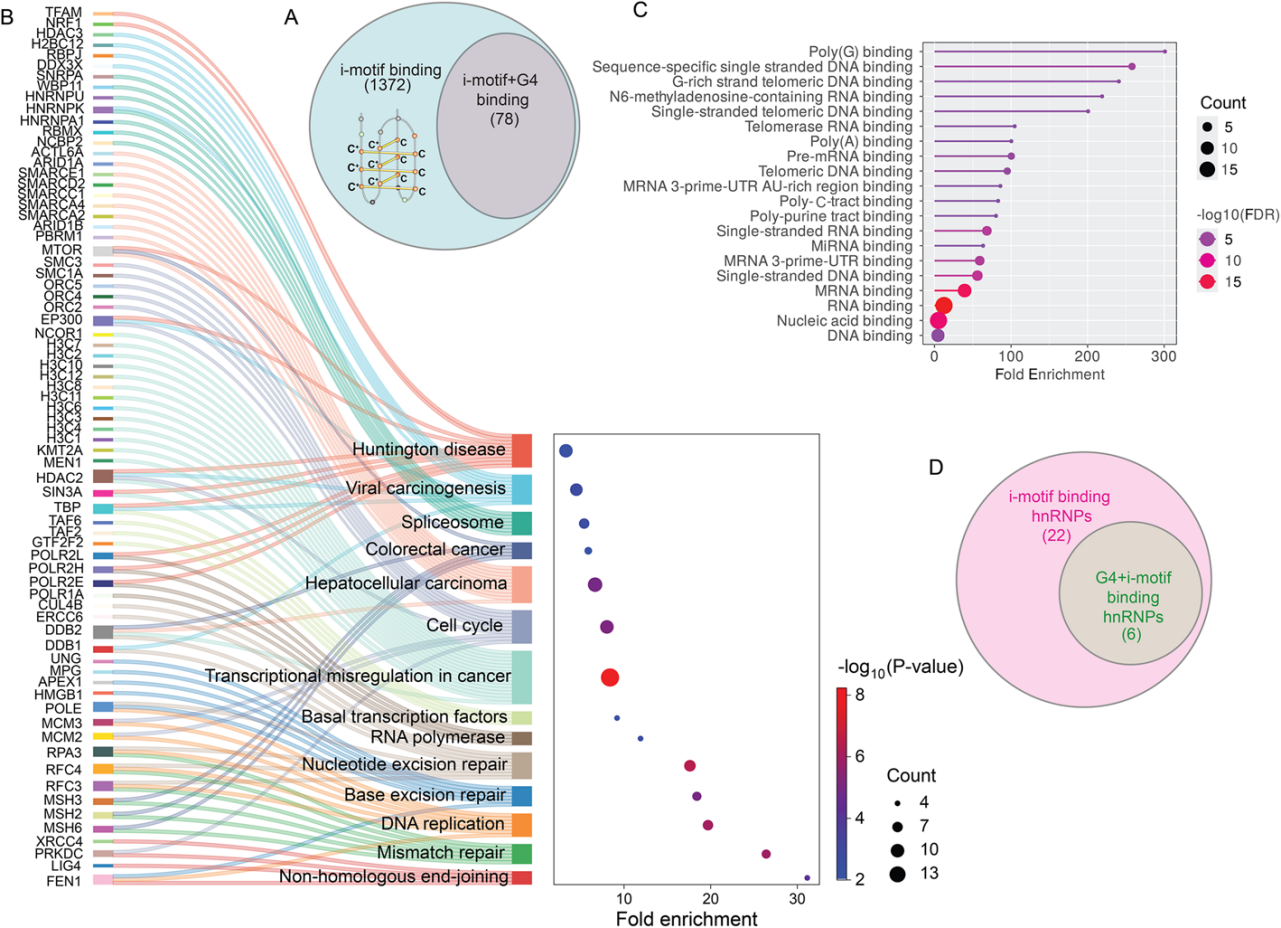
Protein interactions with i-motif structures. **A** Venn diagram. Proteomic studies revealed many candidates that exclusively bind to i-motifs while some bind to both i-motif and G4 structures. **B** Sankey plot showing the GO (Gene ontology) analyses (biological processes) of the proteomics-derived i-motif binding protein candidates and how different proteins are involved or regulate overlapping biological processes. Fold enrichment in x-axis denotes fold increase in protein abundance in i-motif sequence compared to mutated sequence. **C** GO analyses of hnRNP family proteins derived from proteomics data and their sequence-bias. **D** Venn diagram showing number of hnRNP proteins involved in i-motif binding while few hnRNP proteins also interact with G4 structures

Numerous proteins and TFs that are reported to interact with i-motif DNA structures are commonly identified through in vitro binding assays [41, 111, 112] or pull-down-based mass spectrometry [93] or large-scale proteomic screens [106] (Fig. 4A). A substantial number of these proteins are functionally enriched in pathways related to cell cycle regulation, DNA repair, and nucleic acid metabolism (Fig. 4B). Interestingly, several proteins exhibit dual affinities for both G4s and i-motifs (Fig. 4A) (*e.g.*, nucleolin) [106, 113], a property often conferred by multiple nucleic acid-binding domains, including RNA recognition motifs (RRM), zinc-fingers, RGG-boxes, and/or helicase domains. For example, hnRNP-A1 and nucleolin use RRMs to bind both structures, while the helicase BLM can unwind them [112, 114].

Some proteins exhibit conditional binding depending on the local microenvironment. For example, Replication Protein A (RPA) binds G4s at neutral pH and i-motifs more efficiently under acidic conditions, with smFRET studies showing that it unwinds i-motifs even more effectively than G4s [112, 115]. Phase-specific interactions may occur, such as G4 engagement during the S-phase, when replication stress is high, and i-motif binding during early G_1_. RPA accumulates at transient ssDNA during the G_1_/S transition, particularly at replication origins [116, 117]. Similarly, Pif1 family helicases engage G4s during S phase to relieve replication stress, with stabilized G4s recruiting Pif1 to DNA damage foci marked by γH2AX [118–122].

Dynamic protein localization adds another layer of regulation. Members of the heterogeneous nuclear ribonucleoproteins (hnRNP) family undergo nucleocytoplasmic shuttling using nuclear localization sequences (NLS) or M9 motifs in a cell cycle-dependent manner [123, 124]. hnRNP-C resides in the nucleus during interphase, but disperses into the cytoplasm during mitosis [125, 126]. Likewise, hnRNP-K accumulates in the cytoplasm during late G_2_/M, and resides within nuclear compartment during interphase [127]. These shifts likely modulate their access to non-canonical DNA structures. In our recent unpublished study, we demonstrated that hnRNP-E1 (PCBP1) unfolds selective i-motif structures in the genome during G_1_/S transition [128]. Collectively, the hnRNPs emerge as key regulators of non-canonical DNA, yet how their trafficking coordinates with the folding dynamics of i-motifs across the cell cycle remains largely unexplored (Fig. 4C, D).

### hnRNP family proteins

#### hnRNP-K

A well‑studied member of this family, hnRNP-K, contains three K‑homology (KH) domains (KH1–KH3), which bind to RNA or ssDNA, while an additional KI segment between KH2 and KH3 serves as a docking site for protein–protein interactions through proline‑rich motifs. In addition, hnRNP-K contains an NLS that enables nucleocytoplasmic shuttling. It is critical for X‑chromosome inactivation through binding to C‑rich motifs in the Xist RNA B-repeat where binding specificity depends on hairpin formation [129–131]. It is the only protein, identified to date that stabilize i-motif structure in HIV LTR promoter under physiological conditions [93]. Moreover, hnRNP-K binds poly(C) tracts within i-motifs, primarily via KH2, and can unfold these structures, thereby modulating transcription of key genes, such as *MYC*, *KRAS* [111, 132].

#### hnRNP-A1

hnRNP-A1 binds to i-motifs, G4s, and RNA through its RGG domain and RRMs [133–136]. The RGG domain, rich in glycine and arginine, mediates interactions with both RNA and DNA G4s, while the RRMs are crucial for recognizing and binding to specific RNA sequences. hnRNP-A1 and its proteolytic fragment UP1 can also unfold G4 structures, facilitating structural remodeling and downstream protein interactions [133]. Its unfolding of i-motifs contributes to *HRAS* regulation [137], while G4 binding influences *KRAS* [133] and *TRA2B* [138, 139].

#### hnRNP-LL

hnRNP-LL binds to both i-motifs via its two RRMs, which preferentially engage i-motif loop regions. These interactions can stabilize the i-motif, leading to transcriptional activation, or destabilize it, potentially repressing transcription (in *BCL2* promoter) [140].

#### hnRNP-E1 (PCBP1)

PCBP1 (poly(C)-binding protein 1) utilizes its KH domains to recognize and bind to C-rich sequences in DNA and RNA. It preferentially binds heavily oxidized RNA near transcription start sites and regulates multiple aspects of RNA metabolism, including translation and stability. PCBP1 colocalizes with i-motif foci in cells, and its knockdown leads to their disappearance [141]. It recruits DHX36 helicase to the complementary G4 sites, facilitating G4 resolution [142]. Our recent work demonstrated that PCBP1 binds to i-motifs with higher affinity than their unfolded forms with unfolding activity dependent on protonation status and hairpin propensity. PCBP1-mediated resolution of i-motifs occurs at the G_1_/S transition, facilitating replication progression in the i-motif-forming regions [128].

## Conclusion and perspectives

The i‑motif has evolved from a structural curiosity to a candidate regulator of genome dynamics. Its ability to adopt transient pH‑dependent four‑stranded conformation underpins its role in transcriptional control and cellular regulation. In this review, we trace the progress made in understanding i‑motif’s structural characteristics and the physiological modulators of stability. We have examined cellular studies employing a range of experimental methods, highlighting their respective advantages, limitations, and interpretative caveats, while also suggesting ways these techniques can be refined to improve our understanding.

Accumulating evidence highlights interactions with diverse protein partners, particularly hnRNPs, which can stabilize or disrupt i-motifs in a context-dependent manner. Yet, an important challenge remains: many hnRNPs also recognize ssDNA, complicating the distinction between sequence-driven and structure-specific binding. Binding outcomes are further influenced by pH, which affects both i-motif stability and protein activity, raising concerns about experimental conditions that may mask or exaggerate structure-specific recognition. To address this, structure-specific assays, such as using mutated or chemically stabilized i-motifs, are needed to disentangle sequence versus structure recognition.

Looking ahead, advances in high-resolution structural techniques, genome-wide mapping in different organisms, and *in-cell* approaches such as NMR and single-molecule spectroscopy will be essential to establish the physiological roles of i-motifs. Systematic studies across physiological and pathological contexts will further clarify their contributions to genome regulation and disease. Ultimately, the spatiotemporal dynamics of i-motif-prone regions may represent an integral component of genome architecture, whose regulation by hnRNPs provides a rich avenue for mechanistic discovery and therapeutic exploration.

## Data Availability

The proteomics dataset of putative i-motif binding proteins is re-analysed in this review and is available in jPOSTrepo under accession JPST003020 (https://repository.jpostdb.org/entry/JPST003020) and via the ProteomeXchange Consortium under accession PXD051188 (https://proteomecentral.proteomexchange.org/cgi/GetDataset?ID=PXD051188).
